# Purification and Characterization of a DNA-Binding Recombinant PREP1:PBX1 Complex

**DOI:** 10.1371/journal.pone.0125789

**Published:** 2015-04-09

**Authors:** Lisa Mathiasen, Chiara Bruckmann, Sebastiano Pasqualato, Francesco Blasi

**Affiliations:** 1 FIRC (Foundation for Italian Cancer Research) Institute of Molecular Oncology (IFOM), via Adamello 16, 20139, Milan, Italy; 2 Crystallography Unit, Department of Experimental Oncology, European Institute of Oncology, Via Adamello 16, Milan, 20139, Italy; University of Naples 2, ITALY

## Abstract

Human PREP1 and PBX1 are homeodomain transcriptional factors, whose biochemical and structural characterization has not yet been fully described. Expression of full-length recombinant PREP1 (47.6 kDa) and PBX1 (46.6 kDa) in *E*. *coli* is difficult because of poor yield, high instability and insufficient purity, in particular for structural studies. We cloned the cDNA of both proteins into a dicistronic vector containing an N-terminal glutathione S-transferase (GST) tag and co-expressed and co-purified a stable PBX1:PREP1 complex. For structural studies, we produced two C-terminally truncated complexes that retain their ability to bind DNA and are more stable than the full-length proteins through various purification steps. Here we report the production of large amounts of soluble and pure recombinant human PBX1:PREP1 complex in an active form capable of binding DNA.

## Introduction

Homeodomain TALE (three amino acids loop extension) proteins constitute a large class of eukaryotic DNA-binding proteins that regulate transcription of a broad range of developmentally important genes [1]. These proteins share a 60 amino acid DNA-binding domain which has been conserved in sequence, structure and mechanism of DNA-binding. While monomeric homeodomain proteins exhibit a limited ability to discriminate between different DNA sequences, their specificity is significantly enhanced through the cooperative binding with other DNA binding partners. PBX1 (pre-B-cell leukemia homeobox 1) [2,3], and PREP1 (PBX-regulating protein 1) also known as PKNOX1 [4] both belong to the TALE family of homeodomain proteins and form a strong and stable DNA-independent complex [5]. PBX1 contains a nuclear localization signal and carries PREP1 into the nucleus while in turn PREP1 prevents PBX1 nuclear export [6]. PREP1 and PBX1 form trimeric complexes with HoxB1 on target enhancers which play an important role in development [7,8].

PBX1 has a dynamic subcellular localisation. It contains two nuclear localisation signals very close to the homeodomain [6,9] and two nuclear export signals (NES) within the PBC-A domain. Deletion of these Leu/Ile-rich signals impairs nuclear export, although the two NESs [10] were shown to function independently of each other, as deletion of either one did not impair nuclear export. It was suggested that binding of PREP1 masks the NESs and thereby favours retention into the nucleus [6]. The structural knowledge of these transcription factors is limited to NMR structures of PBX1 homeodomain free in solution and bound to DNA [11–13], the crystallographic structure of HoxB1-PBX1 homeodomains and flanking residues bound to DNA [14], and to the NMR structure of free PREP1 homeodomain (PDB: 1X2N). Very little is known of the interaction between PREP1 and PBX1, except that it is lost when the HR1 and HR2 regions are deleted [5]. The three-dimensional structure of this region is not known, nor are the details of the interaction. This interaction is also important because it does not only occur in PREP1, but also in its homolog MEIS1 that likewise is able to form dimers with PBX1 [15]. PREP1 and MEIS1 share identical HR1 and HR2 regions, which in both cases appear to be required to interact with PBX1. Since the number of proteins involved in these interactions is high (four PBX, two PREP and three MEIS, counting only the full length gene products and none of the known alternatively spliced forms), this surface of interaction is worth exploring.

In many cases, structural exploration is made difficult by inherent structural properties of the proteins, like instability. In this paper we report studies aimed at purifying and characterizing a recombinant DNA-binding PREP1:PBX1 complex, and two stable and DNA-binding carboxy-terminally truncated PBX1:PREP1 complexes.

## Results

### Computational analysis predicts that PBX1 amino- and carboxy-termini are disordered, while PREP1 displays low complexity only in its amino-terminus

Secondary structure predictions were performed by using the JPred3 server [16], a web server that in a protein sequence defines each amino acid residue into either α-helix, β-sheet or random coil secondary structures. Identification of low-complexity regions was done using a computer algorithm implemented by the program SEG [17]. This program reports regions of low complexity if there is a continuous stretch of a sequence with an entropy score below a defined threshold. Results from JPred and SEG for PREP1 and PBX1 are summarised in S1A and S1B Fig. PREP1 is predicted to be composed of α-helices and random coils, without β-strands. The conserved regions, HR1 and HR2, are predicted to be predominantly helical in their structure. The homeodomain is predicted to be composed of three α-helices, of which the third is relatively long, compared to other homeodomains. The non-conserved regions of PREP1 are dominated by random coils and stretches of amino acids of low complexity are found in these regions.

The predicted structural organisation of PBX1 is similar: the PBC-A and PBC-B conserved regions are composed of helices and non-conserved regions are dominated by random coils. The region between PBC-A and PBC-B contains an alanine-rich stretch of low complexity. This region has been suggested to function as a flexible linker in complex formation. The homeodomain of PBX1 is predicted to be composed of three α-helices, however from the available structures of PBX1 we know that the third helix is split in two, forming a turn of a 3_10_ helix and a short fourth helix [14].

The sequences of PREP1 and PBX1 were analyzed with GlobPlot2.1 [18] for prediction of the proteins propensity for order/disorder. The results are shown in S1C and S1D Fig. An increase of the disorder propensity sum indicates disorder, whereas a decrease is indicative of ordered/globular structure of the protein. The N-termini of both PREP1 and PBX1 were predicted to be disordered, in the region including the first 50 residues. The C-terminal region to the homeodomain of PBX1 appeared to be disordered from residue 317 just outside of the homeodomain to the C-terminus of the protein, in agreement with the results in S1B Fig predicting lack of secondary structure in regions outside the homeodomain.

### Limited proteolysis on singly expressed proteins

**Fig 1:**
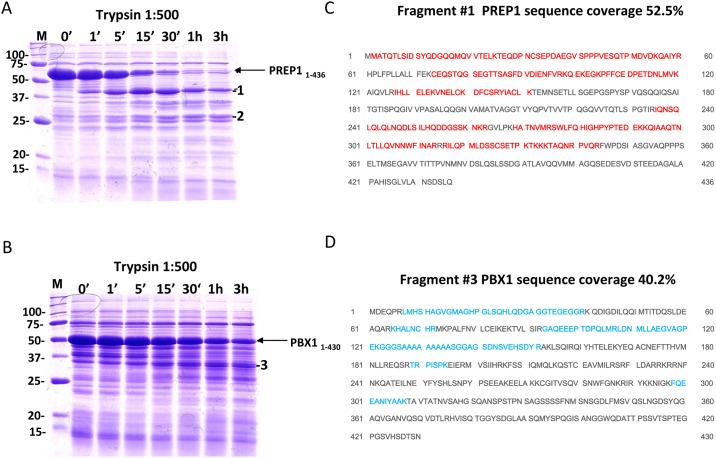
Limited proteolysis analysis of recombinant PREP1 and PBX1. Full length PREP1 and PBX1 were subjected to limited proteolysis with trypsin. The reactions (total volume 100 μl) were performed at room temperature, 10 μl volumes were taken out at the indicated time points, supplemented with sample buffer and boiled prior to loading onto SDS PAGE. The gels were Coomassie stained. **A. Limited proteolysis of PREP1.** Lane M, Bio-Rad size standard; two bands of ~40 kDa (1) and ~28 kDa (2) were chosen for subsequent N-terminal sequencing. **B. Limited proteolysis of PBX1 with trypsin.** Lane M, Bio-Rad size standard; band 3 is the proteolysis fragment chosen for mass spectrometry analysis. **C and D. Identification of PREP1 and PBX1 fragments by MALDI-TOF mass spectrometry analysis.** Peptides of PREP1 and PBX1 were identified by MALDI-TOF analysis after digestion of fragments 1–3 with trypsin. Fragment 1 contained PREP1 and the matching peptides (red) covered 52.5% starting from the N-terminus and ending at residue 344. Fragment 2 contains the N-terminal part of PREP1 excluding the homeodomain. Fragment 3 contains PBX1, and the peptides (blue) covered 40.2% of the sequence, from residue 7 to 308.

We used limited proteolysis, N-terminal amino acid sequencing and mass spectrometry to determine the more stable PREP1 and PBX1 constructs. When a proteolytic cleavage is observed, it usually occurs in non-conserved or disordered regions, the removal of which may improve the stability of the protein. Full-length PREP1 (PREP1_1–436_) and PBX1 (PBX1_1–430_) were subjected to limited proteolysis with trypsin. PREP1 (Fig 1A) was degraded to a distinct ~40 kDa band (band 1) visible on a Coomassie stained SDS PAGE gel. This fragment was stable and persisted after 3 hours of incubation with a 1:500 dilution of trypsin. N-terminal sequencing of this 40 kDa fragment identified the sequence Gly-Pro-Leu-Gly-Ser-Met-Met, corresponding to the PreScission cleavage site in the pGEX6p-2rbs vector and the first two residues of PREP1, indicating that the fragment contained the N-terminus of the protein. Judging from the migration of the fragment, the pattern of trypsin cleavage sites in the PREP1 sequence, and the mass spectromery results (Fig 1D), we estimated that the fragment corresponded to PREP1_1–344_. Therefore, we generated a construct corresponding to this sequence. Another stable fragment of ~28 kDa was visible on SDS PAGE gel (Fig 1A) and was identified in MS as PREP1 fragment 1–230 (band 2). Since this portion of PREP1 does not contain the homeodomain, a DNA construct was not produced.

In the case of PBX1, the largest trypsin-resistant fragment corresponded (Fig 1C) to the region 7–308 (band 3). Since PBX1 contains an arginine at position 6, it is likely that the fragment in fact spans the region 1–308, containing the homeodomain.

Therefore, based on computationally identified disordered region starting at residue 317 and proteolysis data that identified a stable 8–308 fragment, we generated both PBX1_1–308_ and PBX1_1–317_ DNA constructs.

### Purification of the full-length and C-terminally truncated PREP1 and PBX1

Expression and purification of PREP1 and PBX1 during the various steps of purification was evaluated by SDS PAGE and Coomassie staining as shown in Fig 2. Samples from the different purification steps were run in the various lanes as indicated in the figure legend. The theoretical molecular mass of full-length PREP1 is 47.6 kDa, but it runs in SDS PAGE (Fig 2A) with an apparent M_r_ ~ 60 kDa [19]. Full length PREP1 was expressed at relatively low levels <1mg/liter of bacterial culture, whereas the C-terminal deletion mutant PREP1_1–344_ (Fig 2C) was expressed at higher levels as it seemed to be less prone to degradation. Also full length PBX1 showed relatively low expression as compared to its C-terminal deletion mutant PBX1_1–317_ (Fig 2B and 2D). Expression yields are summarized in Table 1.

**Table 1:** Overview of PREP1 and PBX1 constructs.

#	Construct	Molecular weight (kDa)	Yield (mg/L culture)	Comments
1	GST-PREP1(1–436) full-length	47.6	<1 mg	C-terminal degradation
3	GST-PREP1(1–344)	38.2	~1mg	-
13	GST-PBX1(1–430) full-length	46.6	~0.5 mg	C-terminal degradation
15	GST-PBX1(1–317)	35.3	~1 mg	-
16	GST-PBX1(1–308)	34.5	~1 mg	-
25	GST-PREP1(1–436):PBX1(1–430)	47.6 + 46.6	-	Proteins seem highly unstable
35	GST-PBX1(1–430):PREP1(1–436)	46.6 + 47.6	~1 mg	-
38	GST-PBX1(1–317):PREP1(1–344)	35.3 + 38.2	>2 mg	High expression, no degradation
39	GST-PBX1(1–308):PREP1(1–344)	34.5+ 38.2	>2 mg	High expression, no degradation

**Fig 2:**
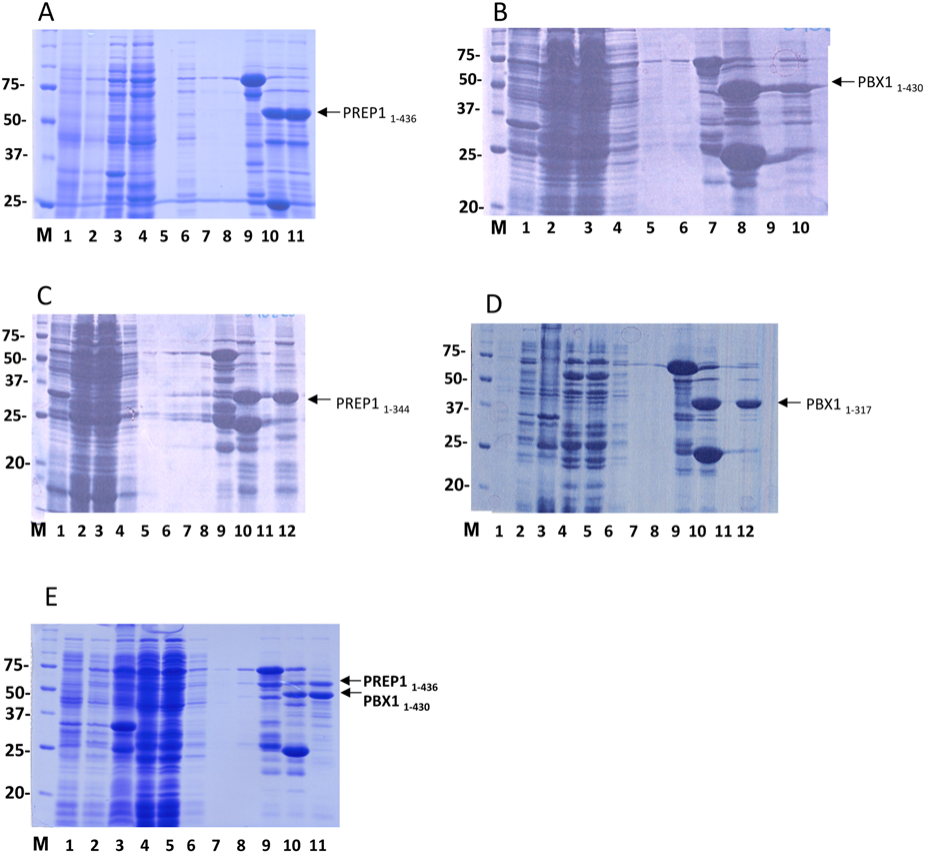
Expression and purification of recombinant PREP1 and PBX1. **A. Expression and purification of full-length PREP1.** SDS PAGE analysis of proteins at different purification stages. Lane M, Bio-Rad size standard; Lane 1, total lysate before IPTG induction; Lane 2, total lysate after IPTG induction; Lane 3, pellet; Lane 4, cleared lysate; Lane 5,empty; Lane 6, first wash after incubation with glutathione beads (Tris-buffer with 0.5 M NaCl); Lane 7, second wash (Tris-buffer with 0.3 M NaCl); Lane 8, third wash (Tris-buffer with 0.3 M NaCl); Lane 9, glutathione beads before elution; Lane 10; glutathione beads after elution; Lane 11, supernatant from glutathione beads, containing the eluted protein. **B. Expression and purification of full-length PBX1.** Lane M, Bio-Rad size standard; Lane 1, total lysate before IPTG induction; Lane 2, total lysate after IPTG induction; Lane 3, cleared lysate; Lane 4, flow-through after incubation with glutathione beads; Lane 5, first wash (Tris-buffer with 0.5 M NaCl); Lane 6, second wash (Tris-buffer with 0.3 M NaCl); Lane 7, glutathione beads before elution; Lane 8; glutathione beads after elution; Lane 9, empty; Lane 10, supernatant from glutathione beads, containing the eluted protein. **C. Expression and purification of PREP1**_**1–344**_. Lane M, Bio-Rad size standard; Lane 1, total lysate before IPTG induction; Lane 2, total lysate after IPTG induction; Lane 3, cleared lysate; Lane 4, flow-through after incubation with glutathione beads; Lane 5, first wash (Tris-buffer with 0.5 M NaCl); Lane 6, second wash (Tris-buffer with 0.3 M NaCl); Lane 7, third wash (Tris-buffer with 0.3 M NaCl); Lane 8, empty; Lane 9; glutathione beads before elution; Lane 10, glutathione beads after elution; Lane 11, empty; Lane 12, supernatant from glutathione beads, containing the eluted protein. **D. Expression and purification of PBX1**_**1–317**_. Lane M, Bio-Rad size standard; Lane 1, total lysate before IPTG induction; Lane 2, total lysate after IPTG induction; Lane 3, pellet; Lane 4, cleared lysate; Lane 5, flow-through after incubation with glutathione beads; Lane 6, first wash (Tris-buffer with 0.5 M NaCl); Lane 7, second wash (Tris-buffer with 0.3 M NaCl); lane 8, third wash (Tris-buffer with 0.3 M NaCl); Lane 9, glutathione beads before elution; Lane 10, glutathione beads after elution; Lane 11, empty; Lane 12, supernatant from glutathione beads, containing the eluted protein. **E. Co-expression and purification of GST-PBX1**_**1–430**_ **PREP1**_**1–436**_. Lane M, Bio-Rad size standard; Lane 1, total lysate before IPTG induction; Lane 2, total lysate after IPTG induction; Lane 3, pellet; Lane 4, cleared lysate; Lane 5, flow-through after incubation with glutathione beads; Lane 6, first wash (Tris-buffer with 0.5 M NaCl); Lane 7, second wash (Tris-buffer with 0.3 M NaCl); Lane 8, third wash (Tris-buffer with 0.3 M NaCl); Lane 9, glutathione beads before elution; Lane 10, glutathione beads after elution; Lane 11, supernatant containing the eluted proteins.

### High-level expression of PREP1:PBX1 complex from dicistronic vector

We then tested the effect of co-expression of PREP1 and PBX1 and its deletion mutants as GST-fusion in the dicistronic expression vector pGEX6p-2rbs. The order in which PREP1 and PBX1 were cloned in the expression vector turned out to be crucial for expression. In the GST-PREP1:PBX1 construct, in which PREP1 is fused to GST, expression was low, whereas PBX1 and PREP1 expressed at significantly higher levels in the construct GST-PBX1:PREP1, where PBX1 is fused to GST (Fig 2E). Expression yields of the various constructs are shown in Table 1.

### Three chromatographic steps for the purification of the PBX1:PREP1 complex

To obtain protein samples of high purity, we employed three chromatographic steps and optimised various parameters in each step. Fractionation was monitored by SDS PAGE followed by Coomassie staining. In order to eliminate heat shock protein 70 and DNA contaminants we added 5 mM MgCl_2_–5 mM ATP in the buffers of the first wash of the glutathione beads and 1 M NaCl in the lysis buffer. The washing buffer used in the last two washes of the glutathione beads had a NaCl concentration of 0.3 M. Therefore, the eluted protein samples were diluted to 0.1 M NaCl for the subsequent ion exchange chromatography step.

**Fig 3:**
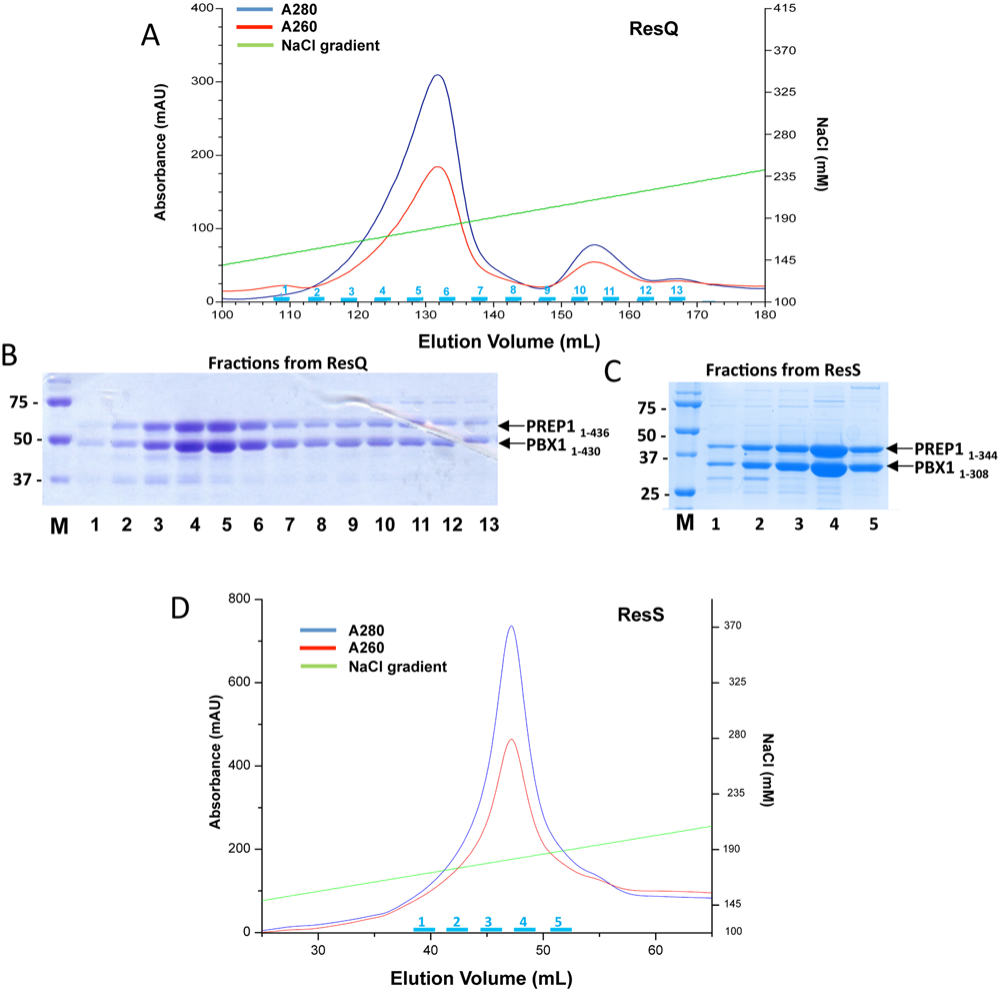
Ion exchange purifications of full-length PBX1:PREP1 and C-terminally truncated PBX1_1–308_:PREP1_1–344_ complexes. **A.** Co-expressed and purified full-length PBX1:PREP1 complex was loaded onto a Res Q anion exchange column and eluted with a 0.1–1 M NaCl gradient. **B.** SDS PAGE of fractions from anion exchange of full-length PBX1:PREP1. Lane M, Bio-Rad size standard; lanes 1–13, fractions indicated in cyan in the above chromatogram; fraction volume was 1.5 ml, and on SDS PAGE were loaded 10 μl of each fraction **C.** SDS PAGE of fractions from anion exchange column of PBX1_1–308_:PREP1_1–344_. Lane M, Bio-Rad size standard; lanes 1–5, fractions indicated in cyan in the chromatogram below; fraction volume was 1.5 ml, and on SDS PAGE were loaded 10 μl of each fraction **D.** Co-expressed and purified PBX1_1–308_:PREP1_1–344_ complex was loaded onto a Res S cation exchange column and eluted with a 0.1–1 M NaCl gradient.

In the case of the full-length PBX1:PREP1 complex, the anion exchange Resource Q column was employed. The majority of the PBX1:PREP1 eluted at 165–200 mM NaCl in a 0.1 M to 1 M NaCl gradient (Fig 3A). A smaller peak which eluted around 210–225 mM NaCl contained a 70 kDa protein contaminant (pointed out in Fig 3B) in SDS PAGE of the fractions.

For the co-purification of the C-terminal deletion mutants PBX1_1–317_:PREP1_1–344_ and PBX1_1–308_:PREP1_1–344_, the cation exchange, Resource S, column was used. PBX1_1–308_:PREP1_1–344_ eluted at 165–200 mM NaCl in a 0.1 M to 1 M NaCl gradient as shown in Fig 3C and 3D, and PBX1_1–317_:PREP1_1–344_ at 220–235 mM NaCl as shown in S2A Fig Material eluting before the main peak contained aggregated proteins, which was evident in the following size exclusion chromatography step and could be avoided only by excluding the first fractions of the main peak from the pool. Fractions from the main peak were pooled and concentrated using Vivaspin concentrators.

**Fig 4:**
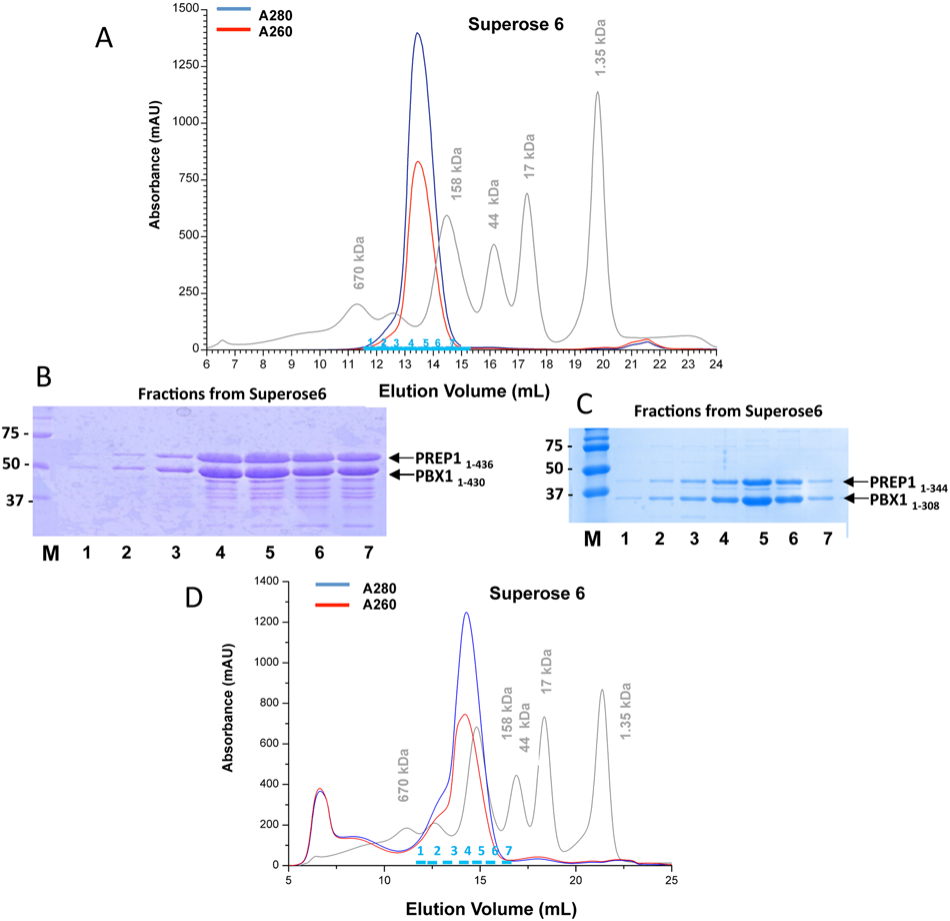
Gel filtrations of full-length PBX1:PREP1 and PBX1 _1–308_:PREP1 _1–344_ complexes. **A.** Size exclusion chromatography on a Superose 6 10/300 column of the full-length PBX1:PREP1 complex purified by anion exchange chromatography. Markers were thyroglobulin (M_r_ 670,000), bovine gamma globulin (M_r_ 158,000), chicken ovalbumin (M_r_ 44,000), equine myoglobin (M_r_ 17,000), and vitamin B_12_ (M_r_ 1,350). **B.** SDS PAGE of fractions from full-length PBX1:PREP1 gel filtration. Lane M, Bio-Rad size standard; lanes 1–7, fractions indicated in cyan in the above chromatogram; fractions volume was 0.5 ml, and on SDS PAGE were loaded 10 μl of each fraction **C.** SDS PAGE of fractions from PBX1 _1–308_:PREP1 _1–344_ gel filtration. Lane M, Bio-Rad size standard; lanes 1–7, fractions indicated in cyan in the chromatogram below; fractions volume was 0.5 ml, and on SDS PAGE were loaded 10 μl of each fraction **D.** Size exclusion chromatography on a Superose 6 10/300 column of the truncated PBX1_1–308_:PREP1_1–344_ complex purified by cation exchange chromatography. Markers were as in panel A.

As a final purification step, the concentrated protein fractions from the ion exchange were loaded onto a Superose 6 10/300 gel filtration column. The full-length PBX1_1–430_:PREP1_1–436_ complex, which has an estimated M_r_ of 94.2 kDa, eluted between 158 and 670 kDa markers, indicating that the complex could be of a higher order stoichiometry (Fig 4A). Fractions from the main peak were pooled and concentrated using Vivaspin concentrators and migrated in SDS PAGE with the expected rate (Fig 4B).

The double C-terminal deletion mutants PBX1_1–317_:PREP1_1–344_, and PBX1_1–308_:PREP1_1–344_ which have an estimated M_r_ of respectively 73.5 kDa and 72.7 kDa, eluted in Superose 6 as a single peaks with an apparent mass of ~200 kDa, again indicating a complex of higher order stoichiometry (Fig 4C,4D and S2C Fig).

**Fig 5:**
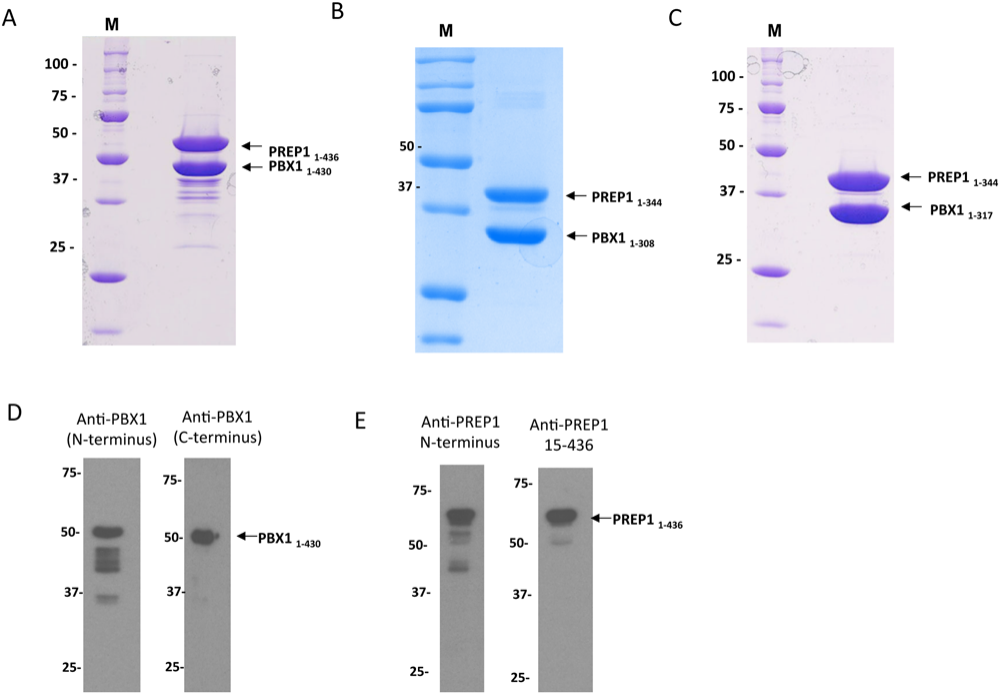
Electrophoretic migration of protein samples after three chromatographic steps. **A, B, and C.** 2 μl of concentrated samples (at 10 mg/ml) of PBX1_1–430_:PREP1_1–436_, PBX1_1–308_:PREP1_1–344_ and PBX1_1–317_:PREP1_1–344_ were loaded onto SDS PAGEs. The full-length proteins are shown in panel A and some degradation products are present. The C-terminal deletion mutants of high purity are shown on the panels B and C. **D and E.** Immunoblots of purified PBX1_1–430_:PREP1_1–436_ complex, where the N-terminal degradation of both PBX1 and PREP1 is evident.

Full length PBX1_1–430_:PREP1_1–436_ complex, the C-terminal deletion PBX1_1–317_:PREP1_1–344_ and PBX1_1–308_:PREP1_1–344_ complexes eluted from Superose 6, were concentrated to 5–10 mg/ml and the SDS PAGE of the samples is shown in Fig 5A,5B and 5C. Fig 5D and 5E shows immunoblottings in which a sample of PBX1_1–430_:PREP1_1–436_ was analysed after purification by SDS PAGE by immunoblotting with anti-PBX1 antibodies directed towards the N-terminus (panel D, left) or the C-terminus (panel D, right). Apart from the band containing the full length PBX1, several bands containing the N-terminal part were present, indicating that degradation/truncation occurs in the C-terminal region of PBX1. PREP1 stability in the PBX1_1–430_:PREP1_1–436_ construct was also analysed by immunoblotting (Fig 5E), using a monoclonal antibody raised against the N-terminus (amino acids 1–155) shown in the panel on the left, and a polyclonal antibody raised against the whole protein (residues 15–436), in the right panel. Degradation of PREP1 is less severe than in PBX1, but we can also observe C-terminal degradation, that leads to a ~40 kDa band.

### Recombinant PBX1:PREP1 complexes are capable of binding DNA

**Fig 6:**
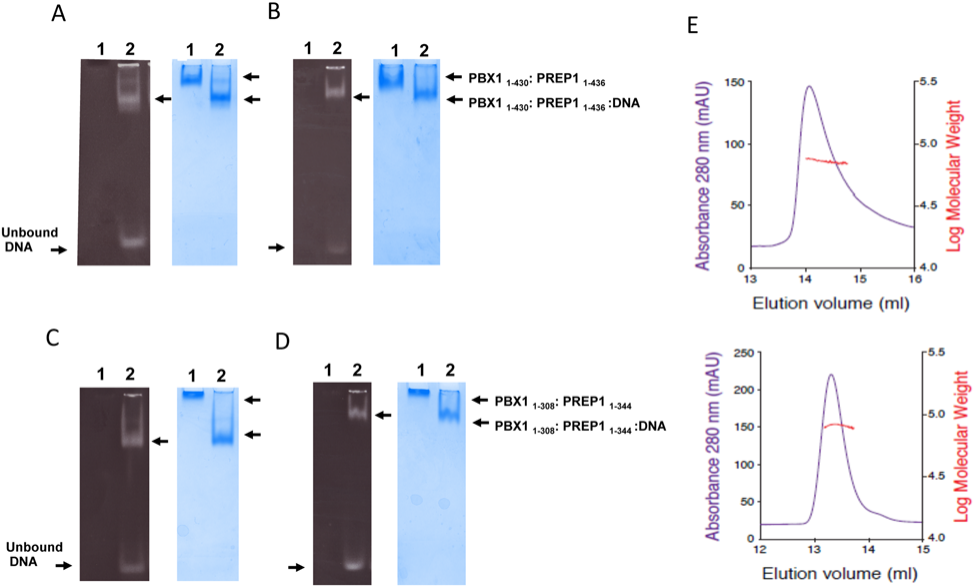
DNA binding of purified recombinant PBX1:PREP1 complexes. **A and B.** EMSA of PBX1:PREP1 complex binding to O1 oligonucleotides. Co-purified full-length PBX1:PREP1 complex (2.5 μM) was incubated in the presence of 1 μg of poly(dIdC) at 1:1 molar ratio with a 22 bp DNA fragment (Panel A) or an 11 bp DNA fragment (Panel B) for 30 minutes at 4°C before loading on gel (Lanes 2). The sequences of DNA are reported in Table 2. As a reference the complex without DNA was also loaded on gel (Lanes 1). Note that with the 22 bp oligo (Panel B) at the highest ratios an additional upper band is visible, possibly corresponding to the binding of a second PREP1:PREP1 complex to the DNA. C and D. EMSA of PBX1_1–308_:PREP1_1–344_:DNA complex. Co-purified PBX1_1–308_:PREP1_1–344_ complex (2.5 μM) was incubated in the presence of 1 μg of poly(dIdC) at 1:1 molar ratio with the 22 bp (Panel C) or the 11 bp DNA fragment (Panel D) for 30 minutes at 4°C before loading on gels (Lanes 2). In Lanes 1 were loaded the protein complexes without DNA. E. Static light scattering analysis of the PBX1_1–308_:PREP1_1–344_ complex. The chromatograms show the UV absorbance in blue (scale on the left) and the calculated molecular mass in red (scale on the right). PBX1_1–308_:PREP1_1–344_ complex was analyzed in the absence (top) and in the presence (bottom) of the 11 bp DNA oligonucleotide

PREP1 was originally identified because it bound to an oligonucleotide corresponding to 31 bp of the Plau gene enhancer (O1, see Table 2); methylation interference studies have indicated that the TGACAG sequence of the human *Urokinase* enhancer was the core binding motif of the PBX1:PREP1 complex [19]. PCR mediated binding site selection *in vitro* and ChIP-seq *in vivo* have identified the motif TGATTGACAG as the optimal binding site for PREP1-PBX1 dimers [20,21] which indeed includes part of the core binding motif TGACAG. We tested the binding of the full length PBX1_1–430_:PREP1_1–436_ complex to two different lengths of the O1 oligonucleotide (Table 2), an 11 bp and a 22 bp fragments, in an electrophoretic mobility shift assay (EMSA). The PBX1:PREP1 complex was incubated at 1:1 molar ratio with the 22 bp (Fig 6A) or 11 bp (Fig 6B) DNA oligonucleotides and in the presence of excess poly(dIdC), loaded onto native gel, alongside a lane with the protein complex without DNA. A band shift was evident in the presence of DNA and only a small amount of unbound DNA was detected in the native gel stained with GelRed (Fig 6A and 6B, left panels), at the bottom of lanes 2. With the 22 bp fragment (Fig 6A), a second minor retarded band formed, visible both with the DNA and Coomassie stainings corresponding to the binding to the DNA fragment of a second PBX1:PREP1 complex, possibly because of a second lower affinity binding site in the 22 bp DNA oligo or to the minor formation of a higher order protein structure.

**Table 2:** O1 oligos.

Oligo	Length	Sequence
O1	21 bp	5’- TCCTGAGGTGACAGAAGGAAG -3’
O1	11 bp	5’- TAGTGACAGAA -3’

It is noticeable that the PBX1:PREP1 complex migrates slower in the absence of DNA than when bound to DNA, as visible in the Coomassie staining (lanes 1 and 2 of Fig 6A and 6B). Furthermore in the presence of 22 bp DNA the migration is greater that with 11 bp DNA. This is easily explainable because in native gels proteins separate on the basis of their charge to mass ratio; when bound to the same protein a 22 bp DNA fragment adds more negative charges to the complex than an 11 bp one.

We also tested on EMSA the ability of C-terminal truncated PBX1_1–308_:PREP1_1–344_ to bind the 11 bp and 22 bp O1 oligos (Table 2) and the results (Fig 6C and 6D) are similar to those obtained for the full-length PBX1:PREP1 complex. Interestingly, the truncated PBX1:PREP1 complex in the absence of DNA does not migrate at all in the gel, due to the high positive charge of the protein complex under the assay conditions. Moreover, the second minor band observed with the full-length complex is absent. This result indicates that deletion of the C-termini does not affect DNA binding.

Overall, the results show that the recombinant complexes retain the DNA binding activity and specificity of the native proteins.

### PBX1_1–308_:PREP1_1–344_ size analysis by static light scattering

The molecular weight of the purified heterodimeric PBX1_1–308_: PREP1_1–344_ complex was determined by static light scattering using the three-detectors method [22]. The chromatograms in Fig 6E show the detector UV readings in blue, while the red line indicates the calculated molecular mass of the two complexes. The panel on top corresponds to the PBX1_1–308_:PREP1_1–344_ apo-complex, while at the bottom is the PBX1_1–308_:PREP1_1–344_:DNA complex_._ Both complexes were monodispersed and displayed a heterodimeric behaviour under these conditions. PBX1_1–308_:PREP1_1–344_ produced an asymmetric single peak eluting at 14.0 ml, and the extrapolated molecular weight (72 kDa) was in perfect agreement with the theoretical molecular weight (72631.2 Da). PBX1_1–308_:PREP1_1–344_:DNA peak eluted symmetrically around 13.3 ml, and indicated an 80 kDa species, consistent with the expected molecular weight of 79302.7 Da.

## Discussion

High-yields of soluble and active proteins are required for structural and biochemical characterizations. Homeodomain transcription factors are not well structurally characterized apart from their DNA binding motif. Expression in *E*. *coli* of sufficient amounts of recombinant full-length homeodomains has surely been a bottleneck, because of their poor yield and insufficient purity.

We singly expressed full-length PREP1 and PBX1, using an N-terminal GST tag to enhance their solubility. The expression was quite low (<1 mg/liter of culture) and we observed C-terminal degradation, resulting in a poor purification yield and low quality. The insertion of both PREP1 and PBX1 in the same dicistronic vector for co-expression and co-purification significantly improved their expression yields. However, PBX1 seems to be more prone to degradation, since we effectively improved the purification yield of the whole complex only by directly fusing PBX1 to the GST tag. Importantly the PBX1:PREP1 complex is resistant to dissociation, because purification steps at high-salt concentration did not affect the stability of the complex. This suggests a hydrophobic nature of the PBX1:PREP1 interaction.

By co-expressing PBX1:PREP1, we increased the purification yield to >1 mg/litre of culture, but the full-length complex, although able to bind DNA as demonstrated by EMSA, was unstable and both PBX1 and PREP1 displayed extensive protein degradation in the C-terminus. Even though we obtained a sufficient amount of protein for biochemical characterization, the purified protein was not homogeneous enough for future structural studies.

Computational analysis predicted that the N-terminal regions of both PREP1 and PBX1 are disordered, while partial proteolysis analysis and western blots pointed out that both recombinant proteins are more prone to C-terminal degradation. PBX1:PREP1 dimerization surface is located in their N-termini, and indeed when the complex is co-expressed N-terminal sequencing of partially trypsinized samples indicated intact N-termini. Instead, PREP1 is sensitive to trypsin proteolysis after residue 344, while PBX1 after residue 308, just downstream of the homeodomains. We cloned into the dicistronic vector the C-terminal truncated forms, including also a PBX1_1–317_ truncated version, according to computational analysis results. Co-expression and co-purification of the C-terminal truncated PBX1:PREP1 increased the yield to >2 mg/liter of culture and we observed no degradation during purification. The deletions end very close to the homeodomains, but the C-terminally truncated PBX1_1–308_:PREP1_1–344_ complex binds DNA as well as the full-length. Interestingly, on gel filtration all the PBX1:PREP1 complexes run with an apparent molecular weight of 200 kDa, twice more than expected. Static light scattering measurements demonstrates that the experimental molecular weight of purified PBX1_1–308_:PREP1_1–344_ complex corresponds exactly to the theoretical one, even when PBX1_1–308_:PREP1_1–344_ is bound to DNA. This discrepancy might be explained because the gel filtration column fractionates proteins on the basis of their hydrodynamic radius (Stokes radius) and not on their molecular weight. The PBX1:PREP1 complex might therefore fold into an elongated complex in solution, or might not have a rigid structure.

Our co-expression and co-purification strategy of PBX1:PREP1 complex, besides improving the expression yields, provides an efficient method for PBX1:PREP1 complex preparation. Proteins produced in this manner are homogeneous and have a purity of >99% on SDS PAGE, ideal for structural studies.

## Materials and Methods

### Protein prediction software

Secondary structure analyses were performed with the web based programs JPred2 and 3 [16], SEG [17] and GlobPlot2.1 [18].

### Cloning of recombinant proteins

Protein constructs were designed by computational sequence analyses aided by limited proteolysis results. The DNA sequence encoding PREP1 contains a BamHI restriction site which was eliminated by QuikChange (Agilent Technologies, Santa Clara, CA) mutagenesis (G→A in position 705) using the primers

PREP_fwd (5’-CCTGGGACAATTAGaATCCAGAACTCCCAGC-3’) and

PREP_bck (5’- GCTGGGAGTTCTGGATtCTAATTGTCCCAGG-3’)

according to the manufacturer’s protocol. DNA encoding full length PREP1 or PBX1, was amplified by PCR with primers containing BamHI and XhoI restrictions sites. Primers used for cloning are shown in Table 3. The amplified PCR products were inserted in pGEX6p-2rbs via BamHI and SalI restriction sites [23]. The mutated DNA was transformed into One-Shot chemically competent TOP10 *E*. *coli* cells (Invitrogen). The protein inserts and the flanking cloning sites were validated by sequencing of the DNA clones.

**Table 3:** Primers used for cloning of PREP1 and PBX1 constructs into PGEX-6p-2rbs.

Oligo	Sequence 5’-3’	Restriction site
PREP1>	cgcggatccATGATGGCTACACAGACATTAAGTATAG	BamHI
PREP1<344	ccgctcgagttaCCTCTGAACTGGCCGGTTC	XhoI
PREP1<436	ccgctcgagCTACTGCAGGGAGTCACTGTTC	XhoI
PBX1>	cgcggatccATGGACGAGCAGCCCAGG	BamHI
PBX1<308	ccgctcgagttaTTTGGCAGCATAAATATTGGCTTC	XhoI
PBX1<317	ccgctcgagttaTGACACATTGGTAGCAGTGAC	XhoI
PBX1<430	ccgctcgagttaTCAGTTGGAGGTATCAGAGTGAAC	XhoI

### Expression Vector

PBX1:PREP1 complex was subcloned in a modified pGEX-6P vector (pGEX-6P-2RBS) to support dicistronic expression. This vector allows cloning of both genes under the control of a single promoter, each gene having its own ribosome-binding site. The same vector was also used for single protein expression.

### Expression and purification of proteins

Expression was performed in the *E*. *coli* strain BL21(DE3)pLysS (Promega, Madison, WI) designed to enhance the expression of eukaryotic proteins that contain codons rarely used in E. coli (i.e. AUA, AGG, AGA, CGG, CUA, CCC and GGA).

Protein expression was induced with 0.1 mM isopropyl-β-D-thiogalactopyranoside (IPTG). Expression was continued for 16–20 h at 16°C. Cells were harvested by centrifugation at 4,000 rpm for 15 minutes in a Beckman JLA rotor and resuspended in lysis buffer (20 mM Tris pH 7.4, 1 M NaCl, 10% glycerol, 0.5 mM EDTA and 1 mM DTT) supplemented with Protease Inhibitor Cocktail Set III Calbiochem (Billerica, MA) and 1 mg/ml lysozyme per litre of *E*. *coli* culture. Sonication was done with a Bandelin Sonopuls (Berlin, Germany) sonicator for 3 × 45 seconds with 5 pulses at 30–40% of max power. After sonication, bacterial lysates were cleared by centrifugation at 40,000 × g for 1–2 hours using a Beckman JA-20 rotor.

### DNA oligonucleotides

DNA oligonucleotides used in this study were purchased from Sigma-Aldrich Biotechnology (Milano, Italy), oligonucleotides labelled with fluorophores were purified by HPLC whereas all other oligonucleotides were purified by desalting. Oligonuclotides for binding studies were annealed by dissolving the lyophilised DNA (0.1–1 mM) in annealing buffer (10 mM Tris pH 7.6, 50 mM NaCl, 0.5 mM EDTA, 5 mM MgCl_2_) and incubating at 95°C in a heat block. After 5–10 minutes the samples were removed from the heating block and allowed to cool slowly at room temperature.

### N-terminal sequencing and mass spectrometry

N-terminal sequencing was performed by automated Edman degradation. Bands for mass spectrometry analysis were cut from the Coomassie stained SDS PAGE gels. Protein samples were digested with trypsin and protein fingerprinting performed by mass spectrometry analysis (MALDI-TOF).

### Antibodies

Anti-PREP1 monoclonal antibody, CH12.2, which was raised against residues 1–155 of human PREP1 was produced by the Antibody and Protein Unit-Cogentech (Milan, Italy). Anti-PREP1 polyclonal raised against the whole protein (residues 15–436) was from Santa Cruz (Dallas, TX) (sc-6245). Anti-PBX1 polyclonal antibody raised against the N-terminal region of human PBX1a was from Cell Signalling (Euroclone, Milan, Italy) (#4342). Anti-PBX1 polyclonal raised against the C-terminus of PBX1/2/3 was from Santa Cruz (sc-888).

### Affinity chromatography

GST-fused proteins were purified using glutathione-sepharose 4B beads (GE Healthcare) according to manufacturer’s instructions. GST was cleaved off with 10 μg/ml of preScission protease (GE Healthcare, Milano, Italy) for 16 hours at 4°C.

### Ion exchange chromatography

GST-free proteins were diluted into ion exchange buffer (20 mM Tris pH 7.4, 0.1 M NaCl, 10% glycerol, 0.5 mM EDTA, 0.5 mM EGTA and 1 mM DTT) to a final NaCl concentration of 0.1 M and run on a Resource Q (GE Healthcare) anion exchange column. Deletion mutants were run on a Resource S (GE Healthcare) cation exchange column. In both cases the proteins were eluted using a 0.1–1.0 M NaCl gradient. The choice of the column was based on the estimated pI values of the proteins.

### Size exclusion chromatography

Proteins were purified by size exclusion chromatography on a Superose 6 10/300 column (GE Healthcare) equilibrated in 20 mM Tris pH 7.4, 0.3 M NaCl, 5% glycerol, 0.5 mM EDTA and 1 mM DTT at a flow rate of 0.3 ml/min. Protein markers used for size exclusion chromatography were the gel filtration standards from Bio-Rad (Hercules, CA). Protein markers used for SDS PAGE were Precision Plus Protein Dual Standards from Bio-Rad.

### Electrophoretic mobility shift assays

Non denaturing gels were prepared as one-step 5–15% gradient in a final volume of 15 ml with 5 or 15% Acrylamide:bisacrylamide solution, 0.8% glycerol, 0,5x TBE (45 mM Tris base, 45 mM boric acid, 1 mM EDTA, pH 8.3), 0.1% ammonium persulfate and 6 μl TEMED (Euroclone). Binding reactions were assembled at room temperature in a total volume of 15 μl (in 20 mM Tris pH 7.4, 150 mM NaCl, 5% glycerol and 1 mM DTT) and incubated 15 minutes before loading. The gel was pre-electrophoresed, at 4°C, for 20 minutes at 90 V. After loading, electrophoresis continued for 2h at 4°C before the gel was stained for 30 minutes with GelRed (Hayward, CA) in 0.5 x TBE for DNA detection. Then the gel was stained in Coomassie Blue for proteins detection. Poly(dIdC) was purchased from Roche Diagnostics S.p.A. (Milano, Italy).

### Limited proteolysis

Trypsin used for limited proteolysis was from Roche Diagnostics. Limited proteolysis was perfomed in 20 mM Tris pH 7.4, 0.3 M NaCl, 10% glycerol, 0.5 mM EDTA and 1 mM DTT at 25°C. The reactions (100 μl) contained 0.150 mg/ml PREP1, PBX1, PREP1:PBX1 complex or PREP1/PBX1/DNA complex. Trypsin, was added to a final concentration of 4 μg/ml or 80 ng/ml and 10 μl volumes were removed at the indicated time points. Reactions were quenched with 10 μl of 5 × SDS PAGE loading buffer, heated at 95°C for 5 minutes, electrophoresed on a 12.5% SDS PAGE gel and stained with Coomassie Blue.

### Static Light Scattering

Static Light Scattering analysis was performed on a Viscotek GPCmax (Malvern, UK) instrument. In our setup, the detectors were connected with two TSKgel G3000PWxl size-exclusion chromatography columns (Tosoh bioscience, King of Prussia, PA) in series. The system was equilibrated in 20 mM Tris buffer pH 7.2, 200 mM NaCl, 5% glycerol, and 1 mM TCEP and calibrated with BSA. PBX1_1–308_:PREP1_1–344_ and PBX1_1–308_:PREP1_1–344_:DNA complexes were both loaded at 1.5mg/ml and eluted isocratically.

## Supporting Information

S1 Fig Secondary structures of PREP1 and PBX1. A. **Secondary sequence prediction of PREP1.** Secondary structure elements predicted by JNet are shown. Random coils are depicted as blue solid lines and α-helices are depicted as blue cylinders. Regions of low complexity as defined by SEG are indicated by an ‘x’. The conserved HR1 and HR2 domains, as well as the homeodomain, HD, are indicated. **B. Secondary sequence predictions of PBX1**. The conserved PBC-A and PBC-B domains, as well as the homeodomain, HD, are indicated. **C and D.** GlobPlot of PREP1 and PBX1. GlobPlot predictions of the disorder propensity for human PREP1a (**C**) and human PBX1a (**D**). The N-termini of both PREP1 and PBX1 are predicted to be disordered. In PBX1, the region C-terminal to the homeodomain appears to be in a disordered state.
(TIF)

S2 Fig Purification of PBX1_1–317_:PREP1_1–344._
**A.** Co-expressed and purified PBX1_1–317_:PREP1_1–344_ complex was loaded onto a Res S cation exchange column and eluted with a 0.1–1 M NaCl gradient. **B.** SDS PAGE of fractions from size exclusion chromatography. Lane M, Bio-Rad size standard; lanes 1–9, fractions indicated in cyan in the chromatogram below; fraction volume was 0.5 ml, and on SDS PAGE were loaded 10 μl of each fraction **C.** Size exclusion chromatography on a Superose 6 10/300 column of the PBX1_1–317_:PREP1_1–344_ complex after cation exchange purification step. Markers were thyroglobulin (M_r_ 670,000), bovine gamma globulin (M_r_ 158,000), chicken ovalbumin (M_r_ 44,000), equine myoglobin (M_r_ 17,000), and vitamin B_12_ (M_r_ 1,350).
(TIF)
