# Supplementary figures and images for: Purification and Characterization of a DNA-Binding Recombinant PREP1:PBX1 Complex
Source: PLoS One. 2015 Apr 9;10(4):e0125789. doi: 10.1371/journal.pone.0125789 (PMC4391845; doi:10.1371/journal.pone.0125789)

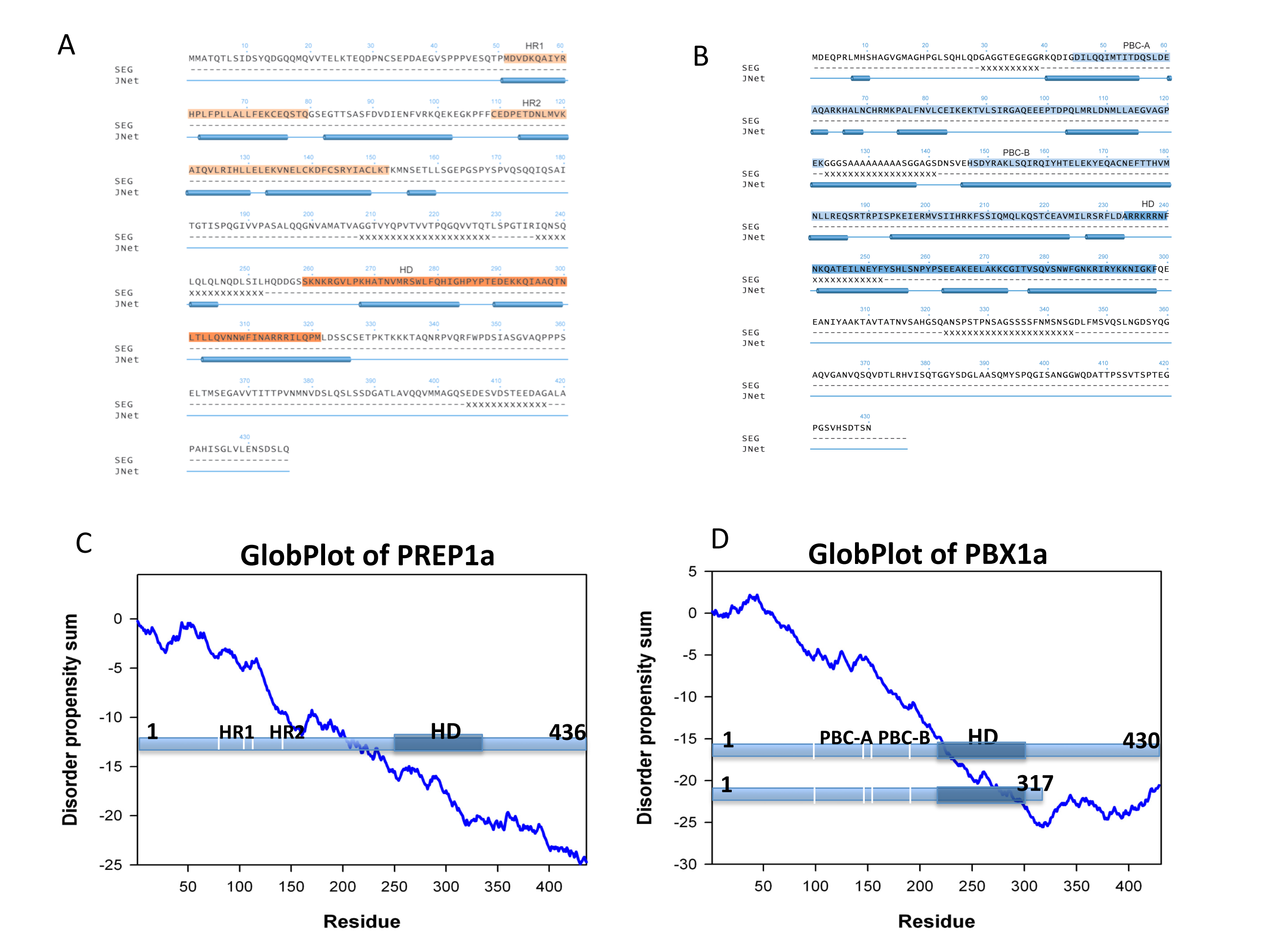

Supplement: S1 Fig — A. Secondary sequence prediction of PREP1. Secondary structure elements predicted by JNet are shown. Random coils are depicted as blue solid lines and α-helices are depicted as blue cylinders. Regions of low complexity as defined by SEG are indicated by an ‘x’. The conserved HR1 and HR2 domains, as well as the homeodomain, HD, are indicated. B. Secondary sequence predictions of PBX1. The conserved PBC-A and PBC-B domains, as well as the homeodomain, HD, are indicated. C and D. GlobPlot of PREP1 and PBX1. GlobPlot predictions of the disorder propensity for human PREP1a (C) and human PBX1a (D). The N-termini of both PREP1 and PBX1 are predicted to be disordered. In PBX1, the region C-terminal to the homeodomain appears to be in a disordered state. (TIF) [file pone.0125789.s001.tif]

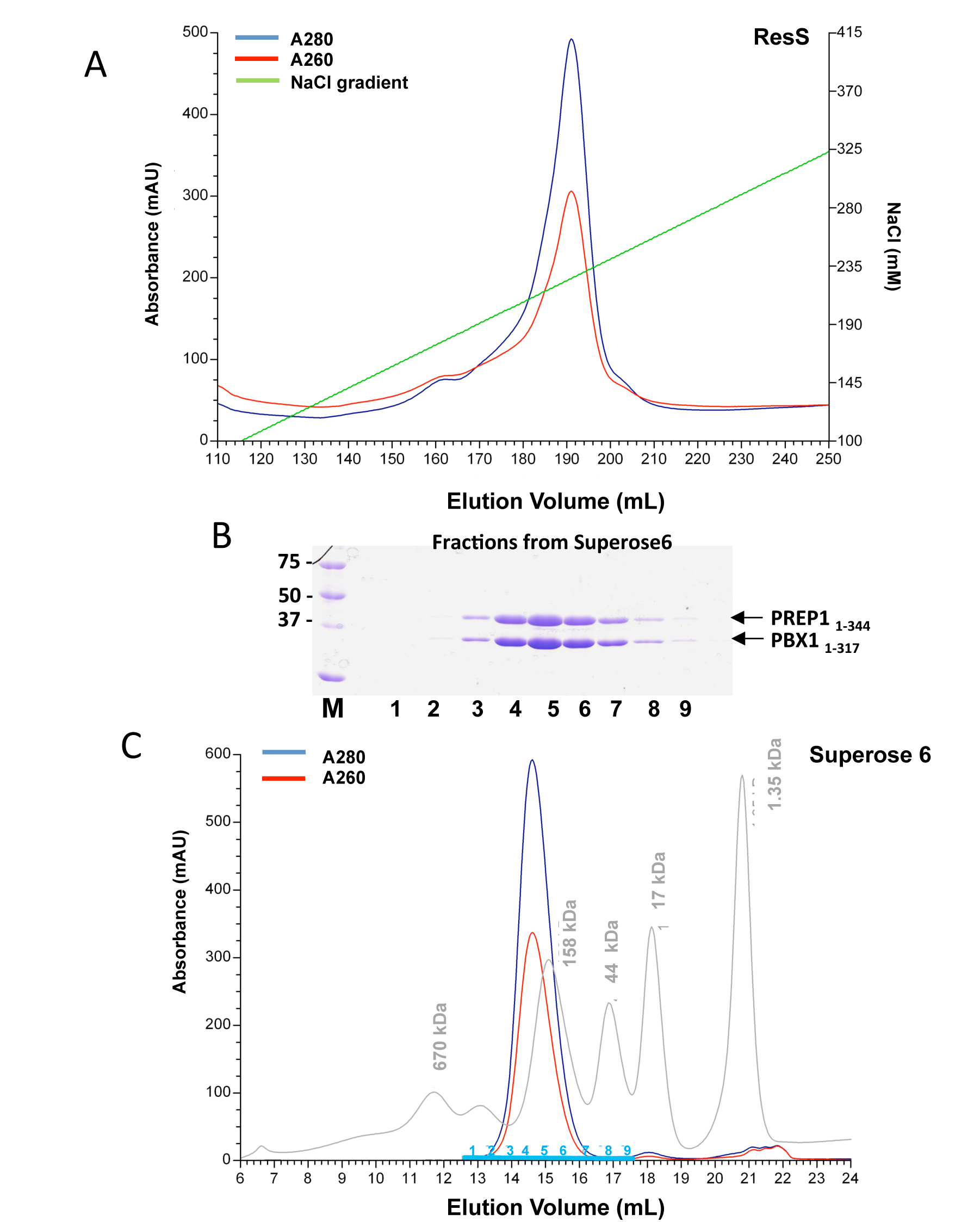

Supplement: S2 Fig — A. Co-expressed and purified PBX11–317:PREP11–344 complex was loaded onto a Res S cation exchange column and eluted with a 0.1–1 M NaCl gradient. B. SDS PAGE of fractions from size exclusion chromatography. Lane M, Bio-Rad size standard; lanes 1–9, fractions indicated in cyan in the chromatogram below; fraction volume was 0.5 ml, and on SDS PAGE were loaded 10 μl of each fraction C. Size exclusion chromatography on a Superose 6 10/300 column of the PBX11–317:PREP11–344 complex after cation exchange purification step. Markers were thyroglobulin (Mr 670,000), bovine gamma globulin (Mr 158,000), chicken ovalbumin (Mr 44,000), equine myoglobin (Mr 17,000), and vitamin B12 (Mr 1,350). (TIF) [file pone.0125789.s002.tif]
